# Combining autophagy and immune characterizations to predict prognosis and therapeutic response in lung adenocarcinoma

**DOI:** 10.3389/fimmu.2022.944378

**Published:** 2022-09-13

**Authors:** Qiaxuan Li, Daipeng Xie, Lintong Yao, Hongrui Qiu, Peimeng You, Jialong Deng, Congsen Li, Weijie Zhan, Maotao Weng, Shaowei Wu, Fasheng Li, Yubo Zhou, Fanjun Zeng, Yong Zheng, Haiyu Zhou

**Affiliations:** ^1^ Department of Thoracic Surgery, Guangdong Provincial People’s Hospital, Guangdong Academy of Medical Sciences, Guangzhou, China; ^2^ Shantou University Medical College, Shantou, China; ^3^ Guangdong Cardiovascular Institute, Guangzhou, China; ^4^ Guangzhou University of Chinese Medicine, Guangzhou, China; ^5^ Department of Thoracic radiology, Cancer Hospital of Nanchang University, Jiangxi Key Laboratory of Translational Cancer Research (Jiangxi Cancer Hospital of Nanchang University), Nanchang, China; ^6^ The Second School of Clinical Medicine, Southern Medical University, Guangzhou, China; ^7^ Department of Cardiothoracic Surgery, Affiliated Hospital of Guangdong Medical University, Guangzhou, China; ^8^ Department of General Practice, Guangdong Provincial Geriatrics Institute, Guangdong Provincial People’s Hospital, Guangdong Academy of Medical Sciences, Guangzhou, China; ^9^ Department of Anesthesiology, Guangdong Provincial People’s Hospital, Guangdong Academy of Medical Sciences, Guangzhou, China; ^10^ Jiangxi Lung Cancer Institute, Nanchang, China

**Keywords:** microenvironment, autophagy, preclinical models, chemotherapy, immunotherapy

## Abstract

**Background:**

Autophagy, a key regulator of programmed cell death, is critical for maintaining the stability of the intracellular environment. Increasing evidence has revealed the clinical importance of interactions between autophagy and immune status in lung adenocarcinoma. The present study evaluated the potential of autophagy-immune-derived biomarkers to predict prognosis and therapeutic response in patients with lung adenocarcinoma.

**Methods:**

Patients from the GSE72094 dataset were randomized 7:3 to a training set and an internal validation set. Three independent cohorts, TCGA, GSE31210, and GSE37745, were used for external verification. Unsupervised hierarchical clustering based on autophagy- and immune-associated genes was used to identify autophagy- and immune-associated molecular patterns, respectively. Significantly prognostic autophagy-immune genes were identified by LASSO analysis and by univariate and multivariate Cox regression analyses. Differences in tumor immune microenvironments, functional pathways, and potential therapeutic responses were investigated to differentiate high-risk and low-risk groups.

**Results:**

High autophagy status and high immune status were associated with improved overall survival. Autophagy and immune subtypes were merged into a two-dimensional index to characterize the combined prognostic classifier, with 535 genes defined as autophagy-immune-related differentially expressed genes (DEGs). Four genes (C4BPA, CD300LG, CD96, and S100P) were identified to construct an autophagy-immune-related prognostic risk model. Survival and receiver operating characteristic (ROC) curve analyses showed that this model was significantly prognostic of survival. Patterns of autophagy and immune genes differed in low- and high-risk patients. Enrichment of most immune infiltrating cells was greater, and the expression of crucial immune checkpoint molecules was higher, in the low-risk group. TIDE and immunotherapy clinical cohort analysis predicted that the low-risk group had more potential responders to immunotherapy. GO, KEGG, and GSEA function analysis identified immune- and autophagy-related pathways. Autophagy inducers were observed in patients in the low-risk group, whereas the high-risk group was sensitive to autophagy inhibitors. The expression of the four genes was assessed in clinical specimens and cell lines.

**Conclusions:**

The autophagy-immune-based gene signature represents a promising tool for risk stratification in patients with lung adenocarcinoma, guiding individualized targeted therapy or immunotherapy.

## Introduction

Lung cancer is one of the most common cancers worldwide with a high mortality rate and poor prognosis. About 85% of patients with lung cancer have non-small cell lung cancer (NSCLC), with the most prevalent histological form of NSCLC being lung adenocarcinoma (LUAD) (1). The prognosis of LUAD patients is still dismal despite advances in diagnosis and treatment, including the introduction of target therapy and immune checkpoint inhibitors (2). As a complex multi-step process, the formation of LUAD may be intimately linked to the expression of various genes (3). Additional molecular indicators are needed to predict the prognosis of patients with LUAD.

Autophagy, a major regulator of programmed cell death, maintains the stability of the intracellular environment by eliminating damaged organelles, misfolded proteins, and pathogens (4). Autophagy has been found to have a key function in various diseases, including infectious diseases, neurodegenerative diseases, and cancer (5). However, the biological mechanism of autophagy in cancer is dependent on cancer type, stage, and other variables (6). Autophagy can suppress tumors by eliminating harmful substances and maintaining genome stability, especially during early stages of tumor growth (7). However, once a tumor has progressed to an advanced stage, autophagy protects tumor cells by enabling them to adapt to hypoxia and nutrient deprivation (8). The overexpression of Beclin1, an essential autophagy gene, is linked to the progression of various tumor types (9). Moreover, autophagy may act as a promoter, enhancing tumor metastasis and aggressiveness (9). Thus, modulation of autophagy has a significant impact on the tumor microenvironment (TME), promoting or inhibiting tumor growth.

Immune cells that infiltrate tumors have been linked to tumor growth, metastasis, and progression (10). The levels of expression of immune checkpoints, such as PD-L1, PD-1, and CTLA-4, play an important role in cancer prognosis and response to immunotherapy (11). Autophagy has been shown to influence the immunological state of the TME, either directly or indirectly (12). Interplay between autophagy and immunity may affect tumor occurrence and development. Autophagy has been reported to be associated with T-cell survival, activation, and effector function (13), whereas tumor autophagy has been shown to weaken NK-cell-mediated tumor cell lysis in a mouse cancer model (14). Additional studies, however, are needed to better understand the mechanism underlying the interactions between autophagy and immunity.

Interactions between autophagy and immunity may have prognostic relevance in patients with LUAD. The present study was designed to create and validate a comprehensive index of molecules and cells associated with immunological and autophagy status that might be used to better describe the TME and predict prognosis in patients with LUAD. This autophagy-immune-related prognostic risk model may be a more accurate prognostic and therapeutic indicator in LUAD.

## Materials and methods

### Data acquisition

All the clinical information and gene expression profiling of patients with LUAD were accessed from The Cancer Genome Atlas (TCGA, https://portal.gdc.cancer.gov/) and Gene Expression Omnibus (GEO, https://www.ncbi.nlm.nih.gov/geo/) databases, including the TCGA-LUAD (*n* = 500), GSE72094 (*n* = 398), GSE31210 (*n* = 226), and GSE37745 (*n* = 106) datasets. **Table 1** shows the clinical baseline characteristics of these datasets in detail. The immunotherapy clinical cohorts included 82 patients with advanced solid tumors (15) and the GSE100797 dataset. Fragments per kilobase per million mapped reads (FPKM) format data were downloaded for the cohorts from the TCGA-LUAD dataset. Autophagy-related genes were obtained from the HADb dataset (http://www.autophagy.lu/), and immune-related genes were obtained from the ImmPort dataset (https://www.immport.org/home). An ethics statement was not required because all of the datasets used in the present investigation were from open-access databases. Genes associated with autophagy, immune signatures, ferroptosis and HLA were included for further analysis in different molecular patterns (**Supplementary Table 1**).

**Table 1:** Characteristic baseline of patients in clinical cohorts.

Variable	GSE72094 (*N* = 398)			TCGA(*N* = 500)	GSE31210(*N* = 226)	GSE37745(*N* = 106)
	Training cohort(*N* = 286)	Testing cohort(*N* = 112)	*p*-value			
**Gender**			0.0572			
Male	118 (41.3)	58 (51.8)		230 (46.0)	105 (46.5)	46 (43.4)
Female	168 (58.7)	54 (48.2)		270 (54.0)	121 (53.5)	60 (56.6)
**Age, years**			0.2509			
≤60 years	52 (18.2)	15 (13.4)		157 (31.4)	108 (47.8)	46 (43.4)
>60 years	234 (81.8)	97 (86.6)		333 (66.6)	118 (52.2)	60 (56.6)
Missing	0 (0.0)	0 (0.0)		10 (2.0)	0 (0.0)	0 (0.0)
**Pathological stage**			0.4585			
Stage I	186 (65.0)	68 (60.7)		269 (53.8)	168 (74.3)	70 (66.0)
Stage II	43 (15.0)	24 (21.4)		125 (25.0)	58 (25.7)	19 (17.9)
Stage III	42 (14.7)	15 (13.4)		81 (16.2)	0 (0.0)	13 (12.4)
Stage IV	12 (4.2)	3 (2.7)		25 (5.0)	0 (0.0)	4 (3.7)
NA	3 (1.1)	2 (1.8)		0 (0.0)	0 (0.0)	0 (0.0)
**KRAS status**			0.6595			
Mutation	98 (34.3)	41 (36.7)				
Wild type	188 (65.7)	71 (63.3)				
**EGFR status**			0.5918			
Mutation	28 (9.8)	13 (11.6)				
Wild type	258 (90.2)	99 (88.4)				
**TP53 status**			0.8551			
Mutation	69 (24.1)	28 (25.0)				
Wild type	217 (75.9)	84 (75.0)				
**Survival status**			0.7665			
Alive	206 (72.0)	79 (70.6)		318 (63.6)	188 (83.2)	29 (27.4)
Dead	80 (28.0)	33 (29.4)		182 (36.4)	38 (16.8)	77 (72.6)

### Identification of autophagy-related and immune-related molecular patterns of LUAD

The k-means machine learning technique was used for unsupervised consensus clustering and to separate samples in the GSE72094 dataset into different molecular patterns based on autophagy- and immune-related genes. Briefly, k-means clustering implemented in the “ConsensusClusterPlus” R package was applied to 1,000 iterations by taking 80% of the samples in each iteration. The optimal number of clusters was determined by the proportional change in the area under the cumulative distribution function (CDF) curves, the consensus matrix heatmap, the proportion of ambiguous clustering (PAC), and the NbClust method, with the number of clusters ranging from 2 to 8 (16, 17). Principal component analysis (PCA) was used to separate diverse subtypes of information in two-dimensional space.

### Validation of the autophagy-immune-related prognostic risk model

Genes overlapping in the intersections of autophagy-, immune-, and autophagy-immune-related DEGs were chosen for univariate Cox regression analyses. Hub genes were filtered using the “glmnet” R package and an autophagy-immune-related prognostic risk model developed using least absolute shrinkage and selection operator (LASSO) analysis and multivariate Cox ratio hazard regression analysis. The risk score of each sample was calculated as:


$$Risk\;score=\overset{n}{\underset{i=1}{\sum }}Coef_{i}\times x_{i}$$

where *Coef*_*i*_ represents the coefficients and *x*_*i*_ represents the expression of each hub gene. Based on their risk scores, patients with LUAD in the TCGA, GSE72094, GSE31210, and GSE37745 cohorts were categorized into high-risk and low-risk subgroups. The sensitivity and specificity of the autophagy-immune-related prognostic risk model were assessed using the “survival” package of R software by applying OS and survival-dependent receiver operating characteristic (ROC) curves.

### Immune cell infiltration

Single sample gene set enrichment analysis (ssGSEA) using R software was utilized to determine the levels of infiltration of subtypes of immune cells, including activated B cells, activated CD4 T cells, activated CD8 T cells, and T follicular helper cells. CIBERSORT and xCell algorithms were utilized to compare differences in the infiltration of 22 and 64 types of immune cells, respectively, based on gene expression profiles among different groups. Tumor purity was assessed using the ESTIMATE algorithm, with ESTIMATE score, immune score, and stromal score determined using the “estimate” package of R software.

### Functional enrichment analysis of autophagy-immune-related prognostic risk model

To investigate possible biological pathways among distinct subtypes, DEGs in the GSE72094 cohort with |log2 fold change (FC)| > 0.5 and an adjusted *p*-value of 0.001 were identified using the “Limma” program. Gene ontology (GO) term enrichment analysis and Kyoto Encyclopedia of Genes and Genomes (KEGG) pathway analysis were performed based on DEGs using R software. In addition, gene set enrichment analysis (GSEA) provided by MsigDB was performed using GSEA, version 4.1.0 (http://www.gsea-msigdb.org/gsea) (18).

### Prediction of tumor chemosensitivity to drugs and potential responses to immunotherapy

Sensitivities to chemotherapeutic agents in the high- and low-risk groups, based on their IC_50_ values, were evaluated using the GDSC database (https://www.cancerrxgene.org/) and the “pRRophetic” package of R software. Potential chemotherapeutic medicines in the CTRP2.0 and PRISM databases were investigated, based on the area under the dose–response curve (SUC) as a measure of drug sensitivity (19). Lower IC_50_ and AUC values were indicative of greater sensitivity to the chemotherapeutic agent. Prospective responses to immunotherapy in the high- and low-risk groups were subsequently compared using the Tumor Immune Dysfunction and Exclusion (TIDE) algorithm.

### Cell culture

A normal lung epithelial cell line (BEAS-2B) and four LUAD cell lines (H1975, HCC827, A549, and PC9) were purchased from the China Center for Type Culture Collection (CCTCC). All cells were maintained in Roswell Park Memorial Institute 1640 (RPMI-1640) medium (Gibco; Thermo Fisher Scientific, Inc.) containing 10% fetal bovine serum (FBS) (Gibco) in a humidified incubator at 37°C and 5% CO_2_.

### qRT-PCR

Seventeen paired LUAD and adjacent normal tissue samples were obtained from Jiangxi Cancer Hospital after gaining ethical approval from the Human Research Ethics Committee of Jiangxi Cancer Hospital (No. 2022ky013). Total RNA was extracted from cell lines and tissue samples using TransZol Up Plus RNA Kits (Transgen Biotech, Beijing, China). A 1.0 mg aliquot of each total RNA sample was reverse transcribed to cDNA using TransScript II One-Step RT-PCR SuperMix. Gene expression was quantified by real-time fluorescent quantitative PCR using SYBR green mixture (Novoprotein) and specific primers (**Supplementary Table 2**) on an ABI Step 1 Plus RT-PCR system (Applied Biosystems, USA). The expression of each target gene relative to that of GADPH was estimated using the 2^−ΔΔCT^ method.

### Statistical methods

The relationships between patient characteristics and overall survival were analyzed by univariate and multivariate Cox regression analyses. The correlations between overall survival and tumor subtypes were calculated using the Kaplan–Meier method with the “survminer” R package. Differences between two groups were compared by two-tailed Student’s *t*-tests or Wilcoxon rank sum tests, as appropriate, whereas differences among three or more groups were compared by the Kruskal–Wallis test. Correlations were analyzed using Pearson’s or Spearman’s correlation methods. R software, version 4.1.0, was used for all analyses, with *p*-values<0.05 defined as statistically significant.

## Results

### Autophagy-associated molecular patterns and autophagy-related DEGs in lung adenocarcinomas

**Figure 1:**
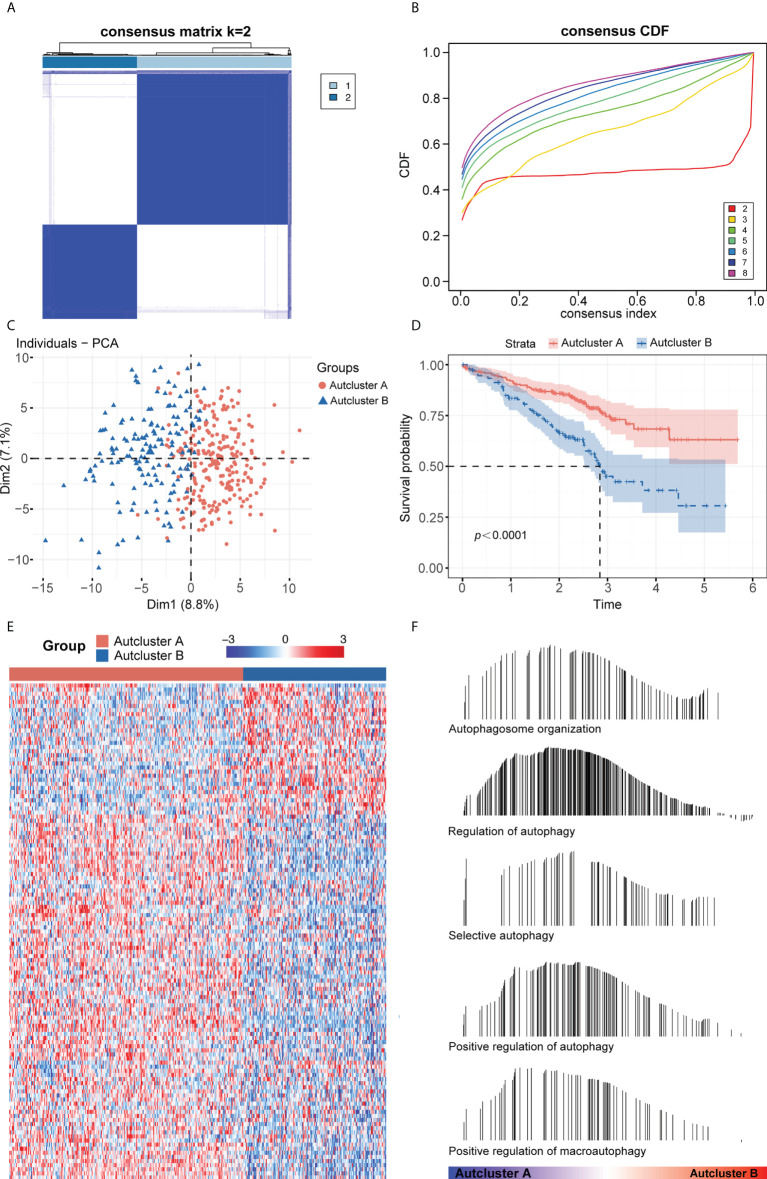
**(A)** Consensus clustering matrix heatmap with autophagy-related molecular pattern in GSE72094 when *k* = 2. **(B)** The CDF curves with *k* valued 2 to 8 (indicated by colors). **(C)** Principal component analysis (PCA) was performed to distinguish AutCluster A and AutCluster B. **(D)** Survival analysis showed better survival among AutCluster A in GSE72094. **(E)** Heatmap of autophagy-related genes in AutCluster A and AutCluster B. **(F)** Autophagy-related pathways enriched in AutCluster A by GSEA.

The intersection of the TCGA-LUAD, GEO, and HADb datasets yielded a total of 208 autophagy-related genes (ARGs), which were utilized to investigate autophagy-related molecular trends in LUAD. Based on ARG expression levels, the R software program ConensusClusterPlus categorized 398 LUAD patients from the GSE72094 cohort into qualitatively different autophagy-associated molecular patterns. The consensus clustering matrix heatmap and the CDF curve indicated that *k* = 2 was optimal (**Figures 1A, B**; **Supplementary Figure 1A**). In addition, the PAC and NbClust algorithms indicated that *k* = 2 was the optimal number for cluster stability (**Supplementary Figures 1B, C**). Thus, two autophagy-associated molecular subtypes were identified, including 247 samples in subtype A and 151 in subtype B. These subtypes were named AutCluster A and AutCluster B, which differed significantly on principal component analysis (**Figure 1C**). Kaplan–Meier analysis showed that survival was significantly better in AutCluster A (*p*< 0.0001, **Figure 1D**). To explore the molecular mechanisms associated with these autophagy-associated subtypes, the expression of ARGs was compared and GSEA enrichment analysis was performed. The levels of most ARGs were significantly higher in AutCluster A than in AutCluster B (**Figure 1E**). GSEA showed that AutCluster A was enriched in autophagy-related pathways, such as those associated with selective autophagy, autophagosome organization, and regulation of autophagy, whereas AutCluster B was enriched mainly in pathways associated with the cell cycle (**Figure 1F**, **Supplementary Table 3**). Overall, patients in AutCluster A and AutCluster B were defined as the autophagy^high^ and autophagy^low^ groups, respectively. In addition, ssGSEA and ESTIMATE analyses showed that the levels of tumor-infiltrating immune cells were higher in the autophagy^high^ than in the autophagy^low^ group (**Supplementary Figures 1D–F**), with GSEA showing that immune-associated pathways were enriched in the autophagy^high^ group (**Supplementary Table 3**). Comparisons of the levels of gene expression in autophagy^high^ and autophagy^low^ groups identified a total of 259 autophagy-related DEGs, with 215 genes overexpressed in the autophagy^high^ group and 44 overexpressed in the autophagy^low^ group (**Supplementary Figure 1G**).

### Immune-associated molecular patterns and immune-related DEGs in lung adenocarcinoma

**Figure 2:**
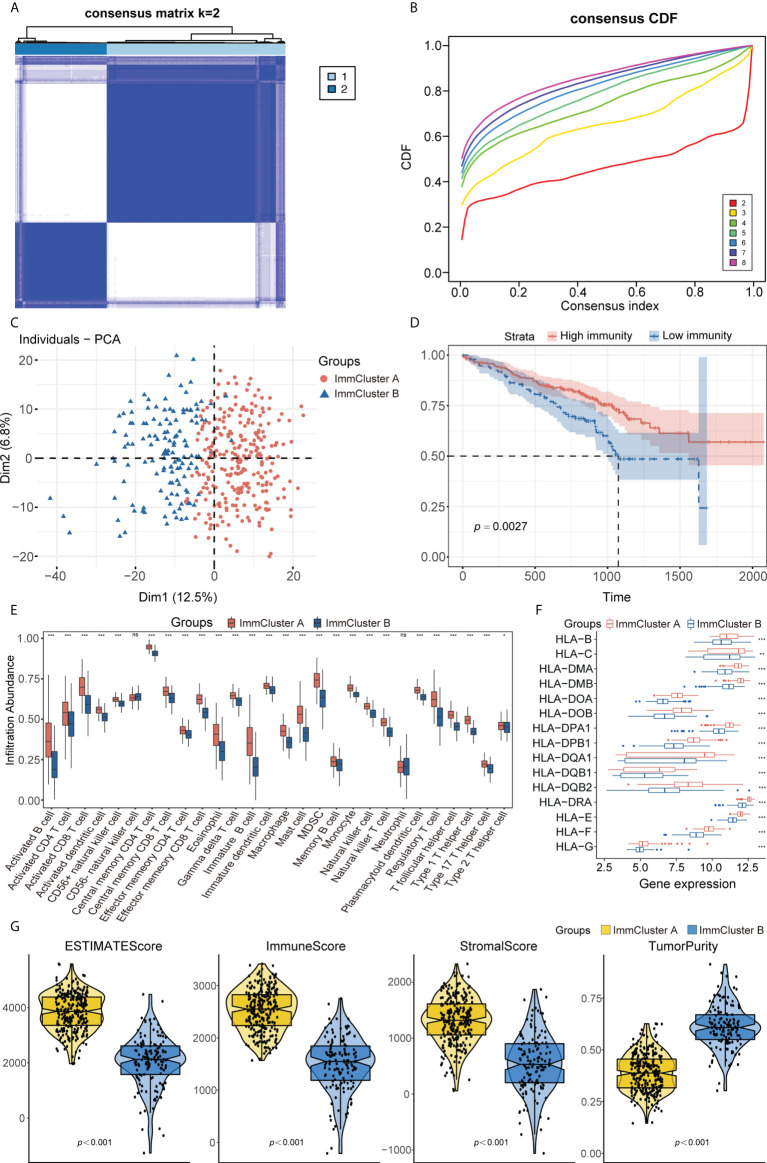
**(A)** Consensus clustering matrix heatmap with immune-related molecular pattern in GSE72094 when *k* = 2. **(B)** The CDF curves with *k* valued 2 to 8 (indicated by colors). **(C)** Principal component analysis (PCA) was performed to distinguish ImmCluster A and ImmCluster B. **(D)** Survival analysis showed better survival among ImmCluster A. **(E)** ssGSEA suggested that ImmCluster A has the higher level of tumor-infiltrating immune cells, such as CD8+ T cell and CD4+ T cell. ns for *p* > 0.05, * for *p* < 0.05, ** for *p* < 0.01, *** for *p* < 0.001. **(F)** Gene expression of HLA gene sets between two distinct clusters. **(G)** Higher immune score, stromal score, estimate score, and lower tumor purity are analyzed in ImmCluster A by ESTIMATE algorithm. ssGSEA, Single sample gene set enrichment analysis.

Combining the genes from the TCGA-LUAD, GEO, and ImmPort datasets yielded 988 immune-related genes that were used to implement consensus clustering in the GSE72094 cohort. Based on the CDF curve of the consensus score, the GSE72094 cohort was divided into two immune-associated subtypes, with 263 patients having subtype A and 135 having subtype B (**Figures 2A, B**; **Supplementary Figure 2A**). Similar results were obtained following PAC and NbClust analyses (**Supplementary Figures 2B, C**), with principal component analysis showing that these two subtypes, defined as ImmCluster A and ImmCluster B, respectively, could be clearly distinguished (**Figure 2C**). Kaplan–Meier analysis showed that patients in ImmCluster A had better survival than those in ImmCluster B (**Figure 2D**). The potential immune landscape of these two subtypes was assessed using ssGSEA and ESTIMATE analyses to investigate their immunologic characteristics. ssGSEA showed that the levels of tumor-infiltrating immune cells, including activated B cells, activated CD8 T cells, effector memory CD8 T cells, activated CD4 T cells, CD56+ natural killer (NK) cells, and T follicular helper cells, were significantly higher in ImmCluster A than in ImmClusterB (**Figure 2E**). CIBERSORT and xCell analysis also showed that the level of adaptive immune cell infiltration was higher in ImmCluster A (**Supplementary Figures 2D, E**). Analysis of the associations between these two immune subtypes and HLA genes showed that the levels of expression of HLA genes were significantly higher in samples from the mmCluster A than the mmCluster B group (**Figure 2F**). Moreover, the ESTIMATE algorithm showed that immune, stromal, and ESTIMATE scores were higher in ImmCluster A than in ImmCluster B, indicating that immune cell infiltration was significantly higher and tumor purity was significantly lower in the ImmCluster A group (*p*< 0.0001 each, **Figure 2G**). Based on these findings, the ImmCluster A and ImmCluster B groups were designated the immunity^high^ and immunity^low^ groups, respectively. A comparison of the immunity^high^ and immunity^low^ groups showed that 365 immune-related DEGs were differentially expressed (**Supplementary Figure 2F**). GSEA showed that autophagy-related pathways were enriched in the immunity^high^ group (**Supplementary Figure 2G**; **Supplementary Table 4**).

### Construction and verification of a combined prognostic classifier and autophagy-immune-related prognostic risk model in lung adenocarcinoma

**Figure 3:**
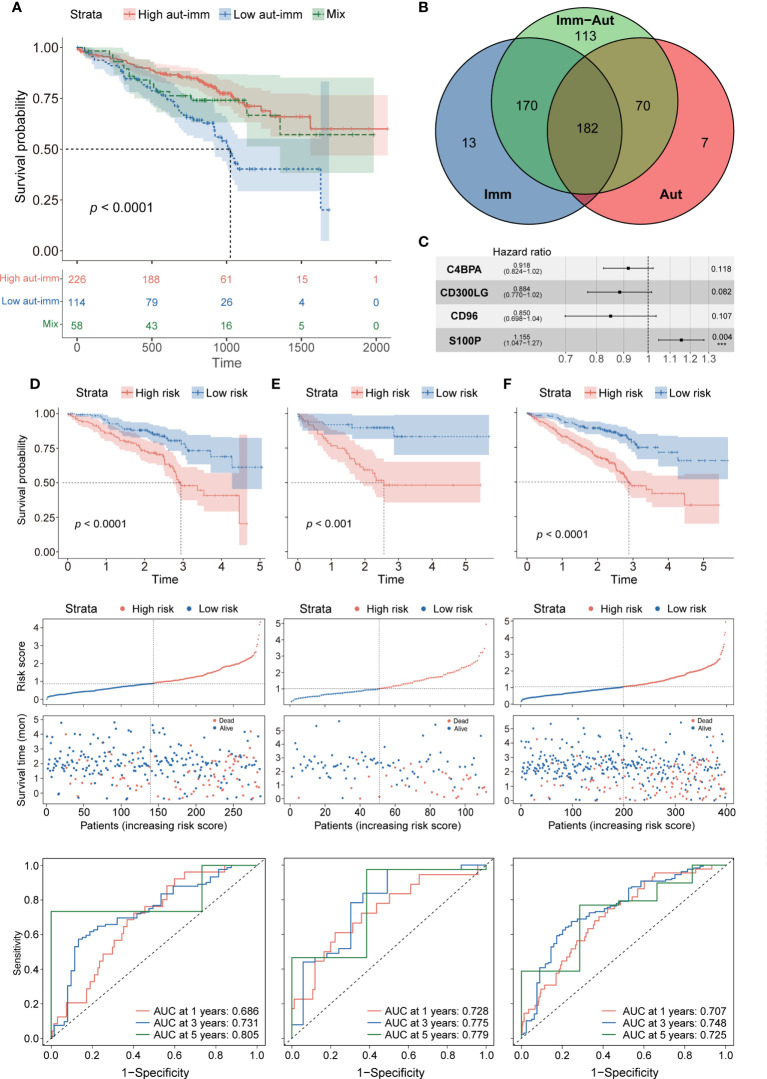
**(A)** Survival analysis between autophagy^high^-immune^high^, autophagy^low^-immune^low^, and mix groups. **(B)** The intersection of autophagy-immune-related genes between different molecular patterns. **(C)** Forest plot of hazard ratios for four autophagy-immune–related prognostic variables. **(D)** The overall survival analysis of the GSE72094 training dataset; construction of the GSE72094 training dataset; the ROC curves at 1, 3, and 5 years of prognostic value of the prognostic in the GSE72094 training dataset. **(E, F)** The validation sets using the GSE72094 testing dataset and the GSE72094 entire dataset.

Autophagy and immune subtypes were merged into a two-dimensional matrix to characterize the combined prognostic classifier. Patients were split into three groups: autophagy^high^-immune^high^, autophagy^low^-immune^low^, and mixed groups (i.e., autophagy^high^-immune^low^ and autophagy^low^-immune^high^). Survival analysis showed that the prognoses were significantly better in the autophagy^high^-immune^high^ and mixed groups than in the autophagy^low^-immune^low^ group (*p*< 0.0001 each, **Figure 3A**). A comparison of gene expression in the autophagy^high^-immune^high^ and autophagy^low^-immune^low^ groups identified 535 DEGs, which were defined as autophagy-immune-related DEGs (**Supplementary Figure 3A**).

The intersection of the autophagy-related, immune-related, and autophagy-immune-related DEGs identified 182 overlapping genes for further investigation (**Figure 3B**). The GSE72094 cohort of 398 patients was divided in a 7:3 ratio into a training set of 286 patients and a testing set of 112. Univariate Cox regression analysis showed that the levels of 24 autophagy-immune-related genes in the training set correlated significantly with OS. LASSO analysis and multivariate Cox ratio hazard regression analysis identified four genes, C4BPA, CD300LG, CD96, and S100P, that were used to construct an autophagy-immune-related prognostic risk model (**Supplementary Figures 3B, C**). The equation for the risk model from these four genes was:

Risk score = (−0.08563) * expression of C4BPA + (−0.12297) * expression of CD300LG + (−0.16202) * expression of CD96 + 0.14384 * expression of S100P (**Figure 3C**).

The relationships of these four genes with tumor-infiltrating immune cells and with essential autophagy-related target genes and the relationships of risk scores with essential autophagy-related target genes and with tumor-infiltrating immune cells were further investigated (**Supplementary Figures 3D–G**). The training set was divided into high-risk and low-risk groups based on the median cutoff value of the prognostic risk grade. Survival analysis showed that OS was significantly longer in the low-risk than in the high-risk group (*p* = 0.00014), and the areas under the curves (AUCs) for 1-, 3-, and 5-year survival were 0.686, 0.731, and 0.805, respectively (**Figure 3D**). Patients in the low-risk group also had more favorable prognoses (*p* = 0.00015), with AUCs for 1-, 3-, and 5-year survival of 0.728, 0.775, and 0.779, respectively (**Figure 3E**). To confirm the reliability of the autophagy-immune-related prognostic risk model, it was used to assess the entire GSE72094 dataset, as well as the TCGA, GSE37745, and GSE31210 cohorts, with similar results observed in all of these cohorts (*p*< 0.0001, **Figure 3F**; **Supplementary Figures 4A–C**).

### Clinical value of autophagy-immune-related prognostic risk model

**Figure 4:**
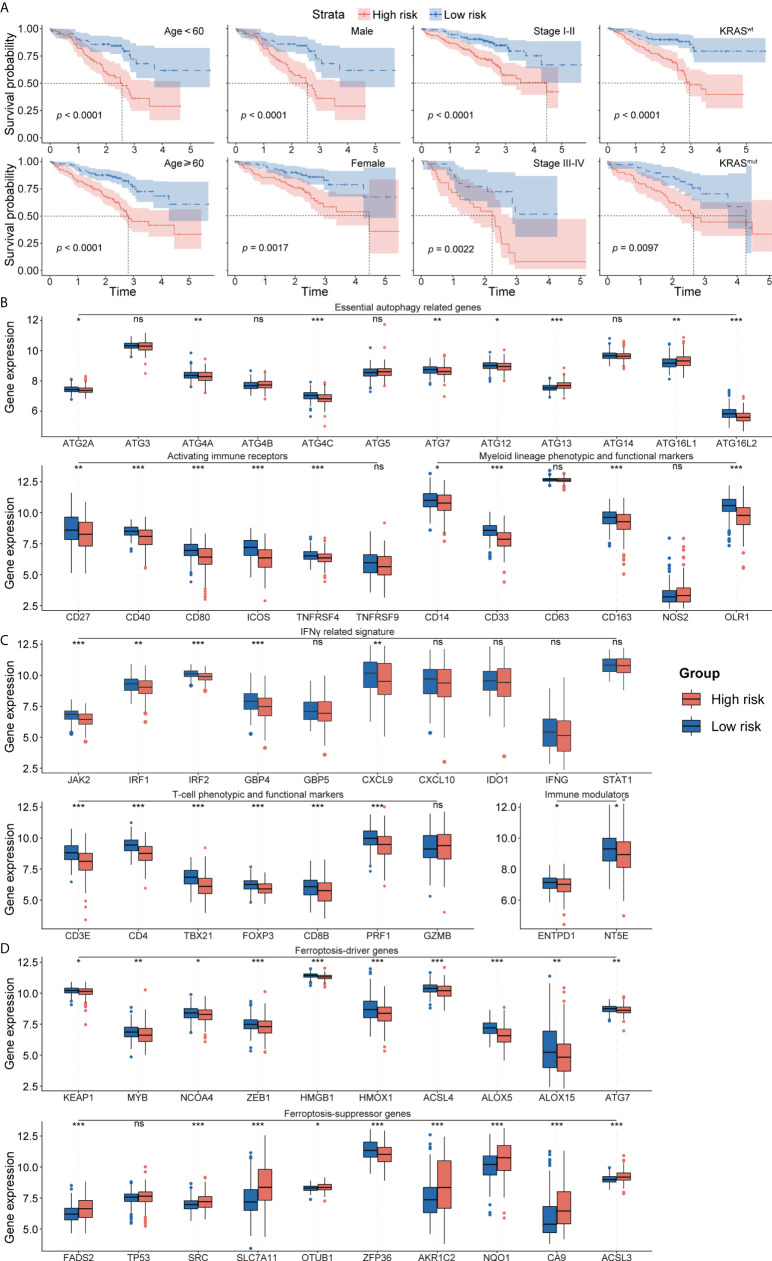
**(A)** Survival analysis showed favorable survival for low-risk patients in different age, gender, pathological stage, and KRAS status. **(B)** The expression of essential autophagy-related genes between the low- and high-risk group. **(C)** The expression of immune response-related genes between the low- and high-risk group. **(D)** The expression of ferroptosis-driver and ferroptosis-suppressor genes between the low- and high-risk group. ns for *p* > 0.05, * for *p* < 0.05, ** for *p* < 0.01, *** for *p* < 0.001.

The prognostic abilities of the autophagy-immune-related prognostic risk model and of many clinicopathological characteristics were investigated using univariate Cox regression analyses. Factors significantly prognostic for OS in the GSE72094 dataset included risk score based on the four autophagy-immune-related genes, as well as patient gender and tumor TNM stage, KRAS status, and EGFR status. Multivariate analysis showed that risk score, gender, and TNM stage were independently associated with patient prognosis (**Table 2**). Patients in the GSE72094 dataset were subsequently stratified by the clinicopathologic characteristics differentiating the high-risk and low-risk groups, including gender (male vs. female), age (<60 vs. ≥60 years), tumor stage (I–II vs. III–IV), KRAS status (wild type vs. mutant), and EGFR status (wild type vs. mutant), and OS was compared in these two groups. Because the number of patients with EGFR mutations was relatively small, the difference in prognosis between high- and low-risk groups, as determined by EGFR mutation status, did not achieve statistical significance. Stratification by other characteristics showed that OS was significantly longer in the low-risk than in the high-risk group (**Figure 4A**; **Supplementary Figure 5A**).

**Table 2:** Univariate and multivariate analysis of clinical characteristics in GSE72094.

Variable	Univariate		Multivariate	
	HR (95% CI)	*p*-value	HR (95% CI)	*p*-value
Age (years)	1.395 (0.810–2.403)	0.229		
Gender	1.552 (1.072–2.246)	0.020	1.506 (1.027–2.209)	0.036
Pathological stage				
I	1	–	1	–
II	2.134 (1.324–3.439)	< 0.001	2.335 (1.440–3.786)	< 0.001
III	3.095 (1.927–4.972)	< 0.001	2.685 (1.667–4.326)	< 0.001
IV	3.351 (1.590–7.062)	< 0.001	3.732 (1.754–7.939)	< 0.001
KRAS status	0.6867 (0.472–0.999)	0.049	0.559 (0.265–1.181)	0.127
EGFR status	3.821 (1.408–10.37)	< 0.001	2.553 (0.922–7.072)	0.071
TP53 status	0.8099 (0.964–1.009)	0.239		
Tumor Purity	2.899 (0.8107–10.37)	0.101		
riskScore	2.041 (1.661–2.508)	< 0.001	1.506 (1.027–2.209)	< 0.001

To explore the effects of the autophagy-immune-related prognostic risk model in autophagy and immune groups, the model was evaluated in the autophagy^high^, autophagy^low^, immunity^high^, and immunity^low^ groups. The risk model separated the patients in these four subtypes into high- and low-risk groups, with the low-risk group having a better prognosis. ROC curve analysis showed that the model had better predictive accuracy in the autophagy^high^ and immunity^high^ groups than in the autophagy^low^ and immunity^low^ groups (**Supplementary Figures 5B–E**).

### Exploration of the tumor microenvironment and responses to immune therapy

To further investigate the TME in each endotype, the expression of individual critical autophagy- and immune response-related genes was mapped to the low- and high-risk groups. The expression of essential autophagy-related target genes was found to be higher in the low-risk group (**Figure 4B**). Immune response-related genes also showed a similar pattern of differential expression, including T-cell phenotypic and functional markers, myeloid lineage phenotypic and functional markers, activating immune receptors, immune modulators, and IFNγ signatures (**Figure 4C**). These results suggested that the levels of autophagy and immune cell infiltration were higher in the low-risk group. In addition, the expression of ferroptosis-driver genes was higher and the expression of ferroptosis-suppressor genes was lower in the low-risk group (**Figure 4D**), suggesting that ferroptosis is activated in the low-risk group and suppressed in the high-risk group.

**Figure 5:**
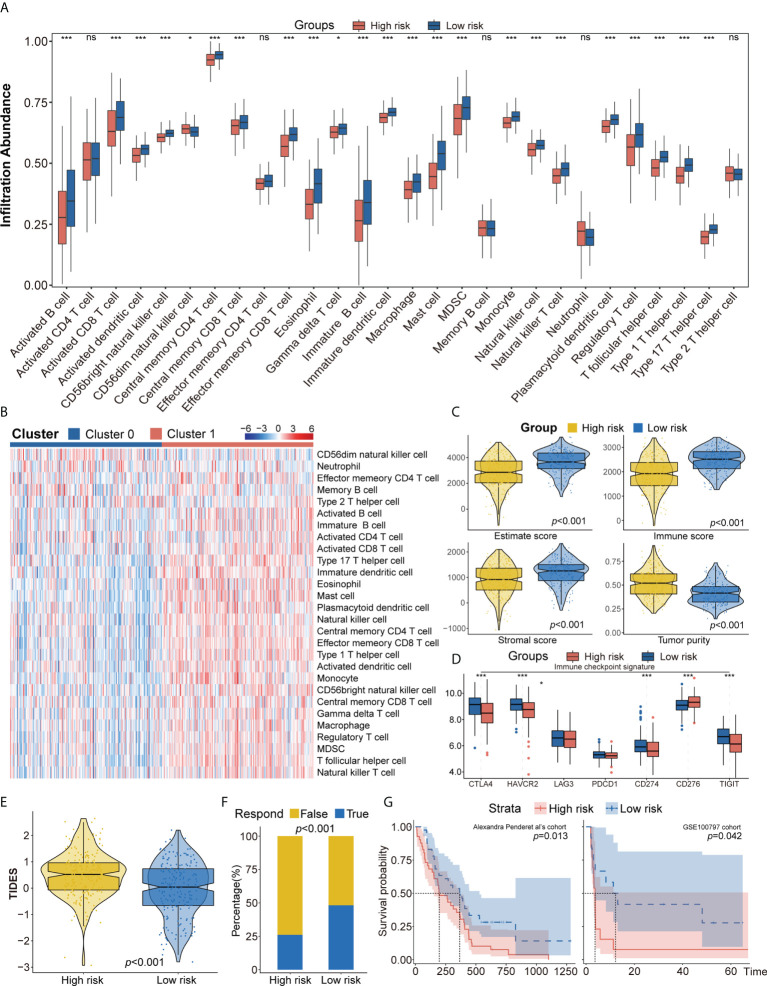
**(A, B)** ssGSEA suggested that the low-risk group has the higher level of tumor-infiltrating immune cells, such as CD8+ T cell and CD4+ T cell. **(C)** Higher immune score, stromal score, estimate score, and lower tumor purity are analyzed in the low-risk group by ESTIMATE algorithm. **(D)** The different expression of common immune checkpoint molecules between the low- and high-risk group, including *PD1*, *PD-L1*, *LAG3*, *CTLA4*, *CD276*, *TIGIT*, and *HAVCR2.* **(E, F)** Estimating T-cell dysfunction and exclusion and predicting response of immunotherapy in the low- and high-risk group by TIDE analysis. **(G)** Describing the model’s efficacy in immunotherapy cohorts by Kaplan–Meier survival analysis. ssGSEA, single sample gene set enrichment analysis; TIDE, Tumor Immune Dysfunction and Exclusion. ns for *p* > 0.05, * for *p *< 0.05, ** for *p *< 0.01, *** for *p *< 0.001.

To further explore differences in the tumor immune microenvironment of high- and low-risk patients with LUAD, their levels of immune cell infiltration were evaluated using ssGSEA, CIBERSORT, and xCell. ssGEA showed that the low-risk group had higher levels of most types of infiltrating immune cells, including activated B cells, activated CD8 T cells, effector memory CD8 T cells, central memory CD4 T cells, CD56+ natural killer cells, immature B cells, mast cells, and T follicular helper cells (**Figures 5A, B**). These findings, along with the results of CIBERSORT and xCell analyses (**Supplementary Figures 6A, B**) showed that the proportion of infiltrating immune cells was higher in the low-risk group than in the high-risk group. Moreover, the ESTIMATE algorithm showed that the low-risk group was positively associated with higher ESTIMTE, immune, and stromal scores, indicating lower tumor purity (*p*< 0.001, **Figure 5C**). The potential responses to immune therapy in the low- and high-risk groups were assessed by comparing their expression of immune checkpoint genes, such as PD1, PD-L1, LAG3, CTLA4, CD276, TIGIT, and HAVCR2. The levels of CTLA4, HAVCR2, PD-L1, and TIGIT were significantly higher in the low-risk group, while the level of CD274 was higher in the high-risk group (**Figure 5D**). The score on the TIDE algorithm, which integrates T-cell dysfunction and exclusion signature to evaluate tumor immune escape, was found to be significantly higher (*p*< 0.001, **Figure 5E**), whereas the predicted proportion of responders was lower (**Figure 5F**), in the high-risk group. We further used Pender et al.’s cohort (immune checkpoint inhibitors to treat advanced solid tumors) and GSE100797 to analyze whether an autophagy-immune-related risk score can predict immune efficacy. The Kaplan–Meier survival analysis shows that the low-risk group has a better survival prognosis than the high-risk group in Pender et al.’s cohort (*p* = 0.013, **Figure 5G**). Analyses of biologic pathways showed that autophagy- and immune-related pathways were enriched in the low-risk group (**Supplementary Figure 6C**). Similar results were observed in the GSE100797 cohort (*p* = 0.042, **Figure 5G**), with patients in this cohort achieving partial response (PR) or complete response (CR) having significantly lower risk scores than those who achieved stable disease (SD) or progressive disease (PD) (*p* = 0.031, **Supplementary Figure 6D**).

### Identification and functional enrichment analysis of autophagy-immune-related prognostic risk model

**Figure 6:**
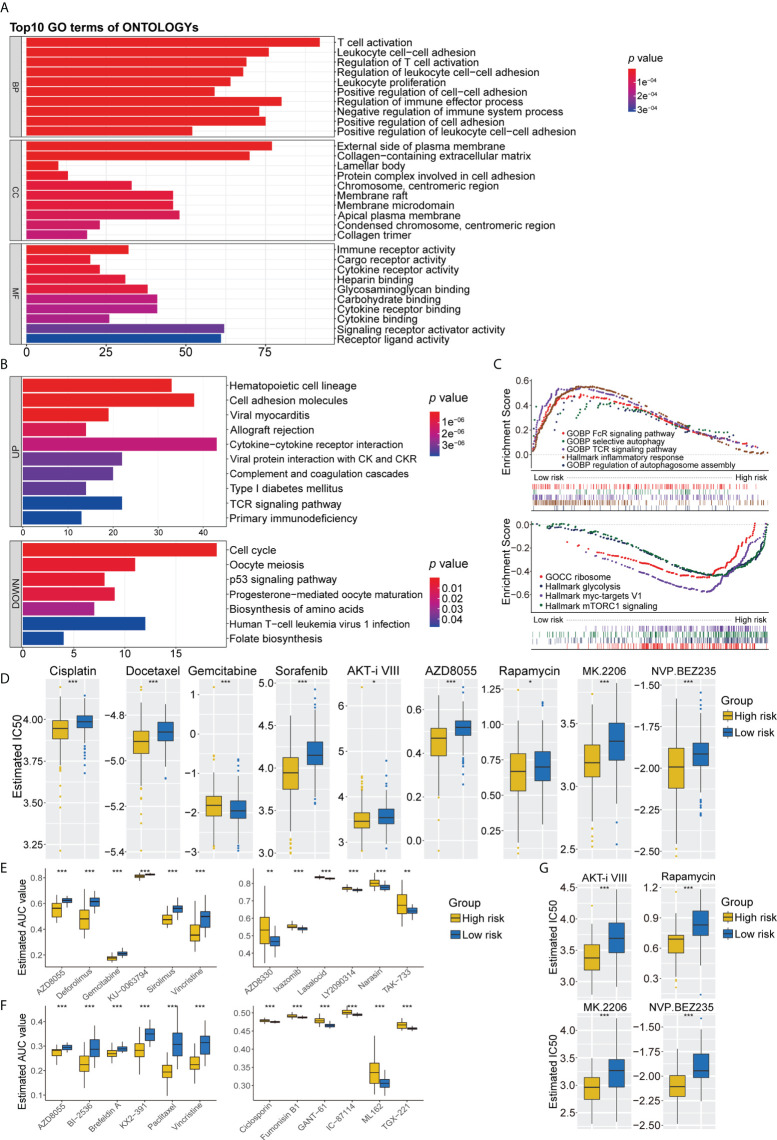
**(A)** GO analysis of differentially expressed genes between the high- and low-risk group. **(B)** KEGG pathways enrichment analysis of differentially expressed genes between the high- and low-risk group; blue represents the high-risk group and red represents the low-risk group. **(C)** GSEA enrichment results of the low-risk group and the high-risk group. **(D)** The results of potential chemotherapy response by the GDSC dataset. **(E)** The results of Spearman’s correlation analysis and differential drug response analysis of 12 PRISM-derived compounds. **(F)** The results of Spearman’s correlation analysis and differential drug response analysis of 12 CTRP-derived compounds. **(G)** The results of potential autophagy inducers in the immunotherapy cohort by the GDSC dataset. GO, Gene Ontology; KEGG, Kyoto Encyclopedia of Genes and Genomes; GSEA, Gene set enrichment analysis. ns for *p* > 0.05, * for *p *< 0.05, ** for *p *< 0.01, *** for *p *< 0.001.

A comparison of the high- and low-risk groups with the “Limma” package, using the criteria *p*< 0.05 and log (fold change) > 0.5, identified 1,342 DEGs (**Supplementary Figure 6E**). GO functional enrichment analysis showed significant enrichment of DEGs associated with T-cell activation, immune receptor activity, chromosomal region, and glycosaminoglycan binding (**Figure 6A**). KEGG pathway analysis indicated that DEGs associated with cell adhesion molecules, cytokine receptor interaction, and T-cell receptor signaling pathways were enriched in the low-risk group, whereas DEGs associated with the cell cycle and metabolic pathways were upregulated in the high-risk group (**Figure 6B**). GSEA pathway enrichment analysis showed that several autophagy-related pathways, such as selective autophagy, positive regulation of autophagosome, and regulation of autophagy and autophagosome organizational pathways, were enriched in the low-risk group, whereas the mTORC1 signaling pathway was upregulated in the high-risk group (**Figure 6C**, **Supplementary Table 5**). These findings indicated that the low-risk group had higher levels of immune cell infiltration and autophagy, as well as higher levels of lysosomes and higher levels of expression of genes associated with Fc epsilon receptor signaling, T-cell receptor signaling, Toll-like receptor signaling pathway, IL6_JAK_STAT3 signaling, apoptosis, interferon-γ responses, and inflammatory responses (**Figure 6C**, **Supplementary Table 5**). Pathways upregulated in the high-risk group included those associated with MYC targets, DNA replication, cell cycle, ribosomes, and glycolysis (**Figure 6C**, **Supplementary Table 5**).

### Predicting the chemosensitivity of high- and low-risk groups to drugs

Because chemotherapy plays an important role in the treatment of LUAD, three separate drug response databases (GDSC, CTRP, and PRISM) were evaluated to identify possible treatment candidates with high drug sensitivity in high- and low-risk LUAD groups. Determination of the IC_50_ values of tumor cells treated with several targeted anticancer agents from the GDSC dataset showed that the estimated IC_50_ levels of cisplatin and docetaxel were significantly lower in the high-risk group, suggesting that this group was more sensitive to these drugs. Patients in the high-risk group were also found to be sensitive to gemcitabine. In addition, several inducers of autophagy, including sorafenib, AKT inhibitor VIII, AZD8055, rapamycin, MK2206, and NVP BEZ235, were identified as potential chemotherapeutic drugs for patients in the high-risk group (**Figure 6D**). Further assessment of chemotherapy candidates using PRISM and CTRP showed that several inducers of autophagy, such as AZD8055, KU-0063794, and deforolimus, were more suitable for patients in the high-risk group, whereas AZD8330, ixazomib, LY2090314, narasin, YM-155, and TAK-733 were found to be potentially effective in the low-risk group (**Figure 6E**). Six CTRP-derived compounds, namely, AZD8055, brefeldin A, paclitaxel, vincristine, KX2-391, and BI-2536, showed high sensitivity in the high-risk group, whereas fumonisin B1, an autophagy inhibitor, was identified as a potential chemotherapeutic drug in the low-risk group (**Figure 6F**). To further validate the synergistic effects of chemoimmunotherapeutic agents, potential drug responses were assessed in immunotherapy cohorts by GDSC. AKT inhibitor VIII, rapamycin, MK2206, and NVP BEZ235 showed greater sensitivity in patients with poorer prognosis for survival (**Figure 6G**). These findings indicate targeted multichannel combinations of autophagy inducers and immune checkpoint inhibitors may enhance immune responses and increase survival rates.

### Expression level of four autophagy-immune-related genes by qRT-PCR

The expression of the four major autophagy-immune genes was assessed by qRT-PCR in 17 paired LUAD and adjacent normal tissue samples. The level of CD300LG mRNA was found to be significantly higher in normal lung than in LUAD tissue samples (**Supplementary Figure 7A**). Similarly, the levels of expression of CD96 and C4BPA were higher in normal lung, although the differences were not statistically significant (**Supplementary Figures 7B, C**). In contrast to previous findings, however, the present study found that level of expression of S100P mRNA was significantly higher in tumor samples than in adjacent normal tissue (**Supplementary Figure 7D**). To further evaluate the role of S100P in lung cancer, its level of expression was compared in the normal lung epithelial cell line (BEAS-2B) and four LUAD cell lines (H1975, HCC827, A549, and PC9). These findings showed that the levels of S100P were higher in all LUAD cell lines than in normal lung epithelium (**Supplementary Figure 7E**).

## Discussion

Autophagy and the immune status of LUAD have been found to affect tumor progression and patient prognosis. Thus, in developing a prognostic strategy, focusing on a single feature may not be sufficient to classify patients with LUAD. The present study explored the potential role of a classifier based on autophagy and immune expression profiles in determining the prognosis of patients with LUAD.

LASSO and univariate and multivariate Cox regression analyses identified four signature genes that comprised an autophagy-immune-related gene model. Three of these genes, C4BPA, CD96, and CD300LG, were associated with better OS, whereas the fourth, S100P, correlated with poorer prognosis. C4BPA had been identified as a novel serum biomarker for pancreatic ductal adenocarcinoma (PDAC) and breast cancer (20, 21). C4BPA was shown to enhance T cell-mediated antitumor immunity in patients with PDAC by promoting CD8+ cell proliferation (22). Regulation of C4BPA can inhibit the PI3K/Akt/mTOR signaling pathway induced by the overexpression of CADM1, thereby affecting the migration and invasion of ovarian cancer cells (23). C4BPA was also found to regulate NF-κB-dependent apoptosis (24). The present study confirmed that C4BPA was overexpressed in the low-risk group and was associated with a higher proportion of infiltrating immune cells and better prognosis in patients with LUAD, as well as being correlated with apoptosis. The expression of CD96 was shown to be significantly elevated in various cancers and to correlate positively with levels of infiltration of several types of immune cells, including CD8+ T cells, DCs, macrophages, monocytes, NK cells, neutrophils, and Tregs (25). As a novel immune checkpoint, CD96 expression was found to correlate strongly with the levels of expression of several other immune checkpoints, such as PD-1, TIGIT, CTLA-4, and CD266 (26). The TIGIT–CD96–CD266 axis was shown to play an important role in regulating T- and NK-cell functions and potential cancer immunotherapy (27). Blockade of CD96 can enhance PD-1/PD-L1 and anti-TIGIT inhibition, leading to increased tumor regression and greater efficacy of immunotherapy (25). The present study showed that CD96 was highly expressed in the low-risk group and was correlated with greater immune cell infiltration and the expression of other co-inhibitory receptors, such as TIGIT and PD-1. Moreover, the higher expression of CD96 in the low-risk group suggested that this group was more likely to benefit from immunotherapy. The only gene associated with the risk of LUAD in the present study was S100P, which had been identified as associated with the risk of several other types of cancer and in the promotion of metastasis. For example, S100P was found to be significantly associated with early recurrence and poorer clinical outcomes in patients with hepatocellular carcinomas (28). S100P was shown to be highly expressed in pancreatic cancer, adult rhabdomyosarcoma, colorectal cancer, and breast cancer (29–32). S100P was also found to be associated with metastasis and poorer survival in patients with LUAD (33). S100P overexpression was also associated with increased angiogenesis and metastasis in subcutaneous tumor xenograft models (34). In addition, the interaction between Keap1 and Nrf2 was found to suppress LUAD tumor progression by inhibiting the S100P protein (35). Although the present study found that S100P expression was associated with autophagy status, further studies are needed to evaluate the relationship between LUAD progression induced by S100P overexpression and autophagy. The present study also identified several potential anticancer agents for treatment of high-risk and low-risk patients, based on the different levels of expression of the four key genes. Additional studies of the relationships of these four genes with potential chemotherapeutic agents may be useful to guide treatment options for patients with LUAD. Overall, the present results provided valuable information on potential biomarkers to determine molecular mechanisms in LUAD. To our knowledge, this is the first composite signature risk model of immunity and autophagy status in LUAD.

Differences in immune and inflammatory landscapes in the low-risk and high-risk groups were evaluated by analysis of tumor-infiltrating immune cells and by metagene analysis. The low-risk group showed higher proportions of immune invading cells, including activated CD8 T cells, effector memory CD8 T cells, central memory CD4 T cells, M1 macrophages, activated B cells, and T follicular helper cells than the high-risk group. Cytotoxic CD8+ T lymphocytes have been identified as anti-tumor immune cells (36). By directly presenting antigen to T cells, B cells play a critical role in anti-tumor immunological responses (37). ESTIMATE analysis in the present study showed that both immune scores and stromal scores were higher in the low-risk group. These results suggest significant associations between immune landscapes and clinical outcomes in patients with LUAD.

The involvement of autophagy in cancer progression remains unclear, as the expression of autophagy-related genes has been associated with both tumor suppression and tumor enhancement (38). Autophagic death can systemically eliminate pre-malignant cells (39). Defective autophagy can result in the accumulation of damaged organelles and misfolded proteins, which can lead to DNA damage and cancer (40). However, autophagy has the potential to promote tumor growth by facilitating immune evasion and cancer metastasis (41, 42). 5′-AMP-activated protein kinase (AMPK) and mTOR complex 1 (mTORC1) have been identified as key regulatory kinases that affect the autophagy process, with AMPK activating and mTORC1 inhibiting autophagy (13). Based on enrichment of autophagy-related pathways, the low- and high-risk groups in the present study were characterized as having high- and low-autophagy status, respectively. The levels of expression of several core autophagic genes, including ATG3, ATG4A, ATG7, ATG12, ATG16L2, and ULK3, were found to be higher in the low- than in the high-risk group. ATG7, a major component of the CK1α/PTEN/FOXO3a/ATG7 axis, has been shown to be involved in the tumor-suppressive process in early LUAD (43). The expression of other autophagy-related genes, such as ATG13, BECN1, and ULK1, was found to be higher in high-risk groups. ULK1-ATG13 was shown to influence the cell cycle and promote tumor progression (44), with GSEA results in the present study also suggesting that the cell cycle pathway was enriched in the high-risk group. In addition, BECN1 and ULK1 have been shown to promote tumor growth in various cancers (45).

Autophagy occurs in all cellular components of the TME, allowing active interventions in interactions among stromal, immunological, and cancer cells (46). Autophagy was found to be involved in modulating immune cell development and differentiation (12). A greater knowledge of the link between autophagy and the immune system might allow the development of individualized cancer therapy. ssGSEA and ESTIMATE analyses in the present study showed a correlation between autophagy status and immune cell infiltration. GSEA pathway analysis of autophagy-associated DEGs showed enrichment of several immune-related pathways, whereas GSEA of immune-associated DEGs showed enrichment of several autophagy-related pathways. These findings indicated that autophagy status and immune status were associated with each other. Toll-like receptors are important mediators of immune regulation that have been shown to promote the activation of autophagy by upregulating the autophagy receptor p62 (47). In addition, damage-associated molecular patterns (DAMPs) have been found to activate innate immune cells and drive autophagic responses *via* Toll-like receptors (48, 49). The present study found that the Toll-like receptor signaling pathway was more prevalent in the low-risk group with better immunological and autophagy states. Tumor-derived lactate has been shown to suppress FIP200 expression, which is required for autophagosome formation, in tumor-infiltrating T cells, resulting in T-cell apoptosis and attenuation of antitumor immunity (50). PIK3C3, an early key player in autophagy, plays a critical role in regulating T-cell differentiation (51). ATG7 deletion results in autophagy-deficient effector CD4+ T cells, which express low levels of IL-2 and IFN (52). Inhibition of autophagy results in the accumulation of depolarized mitochondria in memory CD8+ T cells, resulting in terminal exhaustion of T-cell functional and epigenetic features (53). In contrast, activation of CD8+ cytotoxic T cells by the major histocompatibility complex (MHC) class I frequently results in an immune response, with intact autophagosomes of dead tumor cells required for MHC class I-mediated cross-presentation to CD8+ T lymphocytes (54). The findings of the present study are in agreement with results showing the roles of autophagy in T-cell survival, differentiation, and function. Moreover, autophagy can influence chemokine expression in tumor cells as well as immune cell migration to the tumor. Kras^G12D^-driven lung cancer cells with defective autophagy exhibit high levels of the proinflammatory chemokine CXCL5 (55).

Ferroptosis has been shown to be an important mechanism by which CD8+ T lymphocytes influence tumor death (56). Autophagy plays an indispensable role in the process of ferroptosis, including in ROS accumulation (57). Thus, activation of autophagy may enhance tumor ferroptosis and increase the effectiveness of tumor immunotherapy. The present study found that the expression of ferroptosis-driver genes was higher in the low-risk group, consistent with results linking increases in autophagy and immunity with increases in ferroptosis. Evaluations of cell metabolism has shown that glycolysis is activated following reduction in autophagy flux, with this being corrected by decreased CD8+ T-cell infiltration (58). The present study found that the glycolysis pathway was more prevalent in the high-risk group, confirming the relationships among glycolysis, autophagy, and immunity. Overall, autophagy appears to change tumor antigen presentation, immune cell survival, and function in the TME.

Because autophagy is required for the modulation of tumor immunity, targeting autophagy in combination with other cancer therapies may enhance patient prognosis. Autophagy is involved in the regulation of immunogenic cell death (ICD), which is important for tumor-specific immunity and anticancer immune responses. In response to ICD inducers, such as oxaliplatin, tumor autophagy can promote the preservation of lysosomal ATP reserves and increase the number of dendritic cells to enhance antitumor immunity (59). Autophagy may induce tumor immunogenicity during radiotherapy (60). In contrast, preclinical studies have found that high autophagic flux may lead to chemotherapy resistance of several cancers, such as NSCLC and bladder cancer (61, 62). A comprehensive understanding of the nature of chemotherapy-induced autophagy will guide the development of future clinical trials.

Because autophagy is involved in tumor-infiltrating immune cell development, inducers of autophagy may enhance the efficacy of immunotherapy. Several preclinical models, however, have shown that the immune system may tolerate a certain level of autophagy suppression (63). The efficacy of autophagy inhibitors in combination with immunotherapy has therefore been evaluated. For example, when combined with ICB therapy, CQ, a traditional inhibitor of autophagy, was found to improve antitumor immune response by preventing autophagy-mediated MHC class I degression (64). In addition, CQ was found to limit the toxicity and enhance the immunotherapeutic efficacy of high-dose IL-2 in a mouse model of metastatic liver cancer (65). Clinical trials testing HCQ in combination with immunotherapy are currently underway in patients with metastatic RCC and pancreatic cancer (13). The present study found that patients in the high-risk group in the GSE72094 dataset, with poorer immunological and autophagy status, were more sensitive to inducers of autophagy, such as AKT inhibitor VIII, AZD8055, rapamycin, MK2206, NVP BEZ235, and KU−0063794, than patients in the low-risk group. Several inducers of autophagy were identified as potential chemotherapeutic drugs for patients in the high-risk group. In contrast, patients in the low-risk group were found to be highly sensitive to fumonisin B1, an inhibitor of ceramide synthesis that interferes with autophagy. These findings suggest the following hypothesis: At lower levels of immune cell infiltration, autophagy plays a protective role in inhibiting tumor growth, with autophagy-inducing agonists improving the prognosis of patients with LUAD. At higher levels of immune cell infiltration, however, autophagy may impair immune function and promote tumor growth, such that autophagy inhibitors can be used to effectively treat these patients. Evaluation of the tumor immune microenvironment is therefore required to determine the optimal patient treatment strategy, which may consist of combinations of immunotherapeutic agents and either inhibitors or inducers of autophagy. Additional studies are needed to fully determine the various functions of autophagy pathways and their possible interactions in tumor immunity and immunotherapy. Combining autophagy and immunity may not only serve to classify patient prognosis but to guide treatment.

The present study had several limitations. First, all analyses were performed on data obtained from public databases. Prospective, multicenter studies are therefore required to validate the generalizability of this model. Second, because the microenvironmental properties of various tumor regions differ, the autophagy and immunological states at distinct tumor locations may not be distinguishable when the tumor is viewed as a whole. In addition, because of the limitation of the drug dataset, many autophagy core drugs, such as HCQ, CQ, and 3-MA, were included in the present study. Additional preclinical and clinical trials are required to verify the effect of combinations of immunotherapeutic agents with autophagy inducers and inhibitors. Third, the numbers of paired clinical specimens and cell lines used to assess gene expression by qRT-PCR may have been too small. The lack of a sufficient number of samples may explain the disparate qRT-PCR results obtained in clinical specimens and cell lines. Finally, because the regulation of autophagy depends on the phosphorylation and dephosphorylation of autophagy-related proteins, the present findings based on RNA sequences do not provide a complete picture of the role of autophagy in LUAD. Proteomic analyses are needed prior to applying these findings to clinical practice.

## Data availability statement

The datasets presented in this study can be found in online repositories. The names of the repository/repositories and accession number(s) can be found in the article/**Supplementary Material**.

## Ethics statement

The studies involving human participants were reviewed and approved by The Human Research Ethics Committee at Jiangxi Cancer Hospital (NO.2022ky013). The patients/participants provided their written informed consent to participate in this study.

## Authors contributions

QL, DX, and LY contributed to conceptualization, data curation, formal analysis, data curation, software and project administration, and writing manuscript. HQ contributed to software, visualization, and manuscript editing. PY and JD contributed to experiment and validation. CL, WZ, and MW contributed to the methodology and resources. YBZ, SW, and FL contributed to manuscript review. HZ, YZ, and FZ contributed to project administration and supervision. All authors contributed to the article and approved the submitted version.

## Funding

This study was funded by the Natural Science Foundation of Guangdong (Grant Number 2021A1515010838), the Science and Technology Program of Guangzhou (Grant Number 201903010028), and the Guangdong Provincial People’s Hospital Intermural Program (Grant Number KJ012019447).

## Acknowledgments

We appreciate the data obtained from TCGA and GEO. We sincerely thank all the people involved.

## Conflict of interest

The authors declare that the research was conducted in the absence of any commercial or financial relationships that could be construed as a potential conflict of interest.

The handling editor JZ declared a shared parent affiliation with the author WZ at the time of review.

## Publisher’s note

All claims expressed in this article are solely those of the authors and do not necessarily represent those of their affiliated organizations, or those of the publisher, the editors and the reviewers. Any product that may be evaluated in this article, or claim that may be made by its manufacturer, is not guaranteed or endorsed by the publisher.
