# Supplementary figures and images for: Combining autophagy and immune characterizations to predict prognosis and therapeutic response in lung adenocarcinoma
Source: Front Immunol. 2022 Sep 13;13:944378. doi: 10.3389/fimmu.2022.944378 (PMC9513242; doi:10.3389/fimmu.2022.944378)

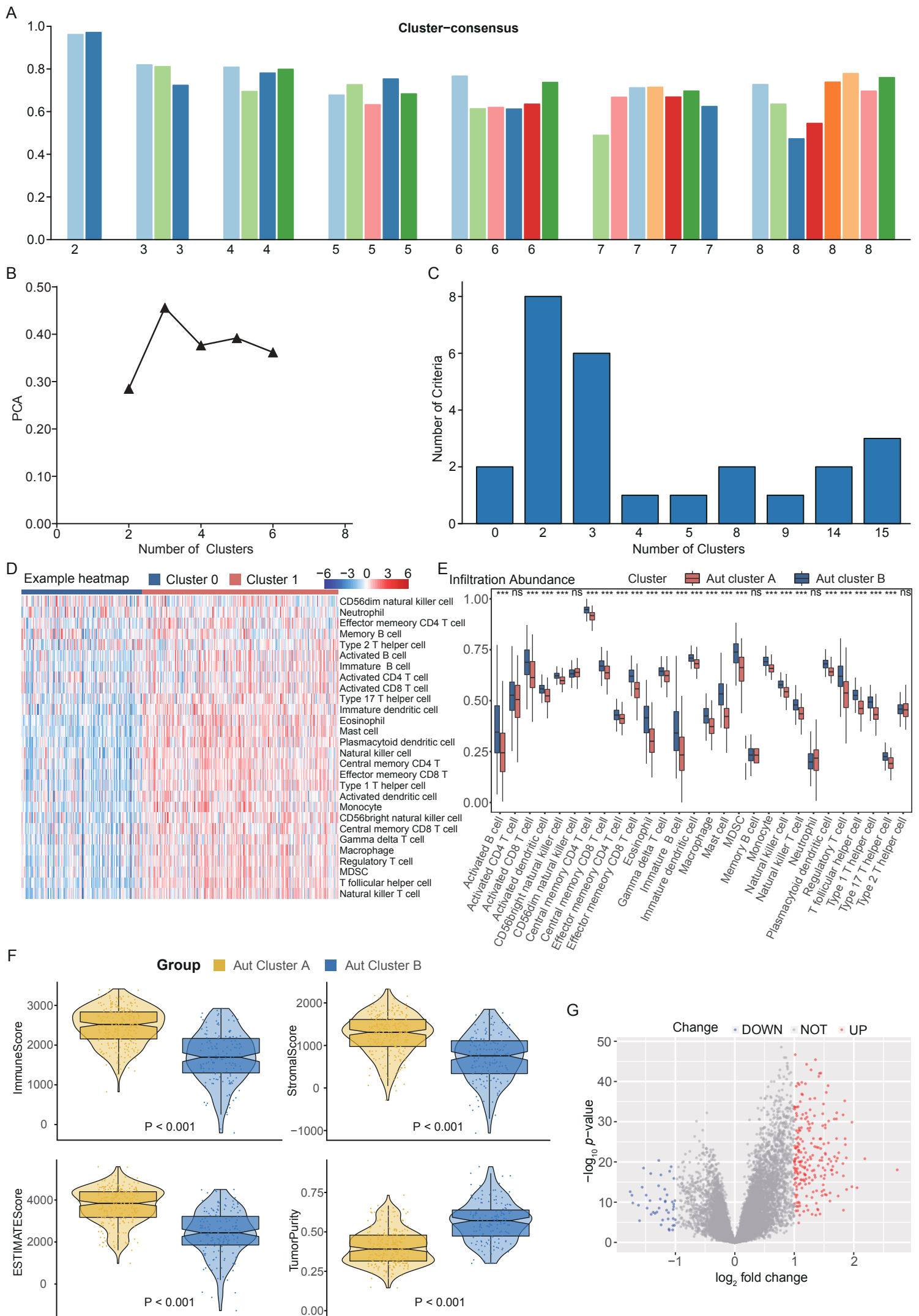

Supplement: Supplementary file 1 [file DataSheet_1.pdf]

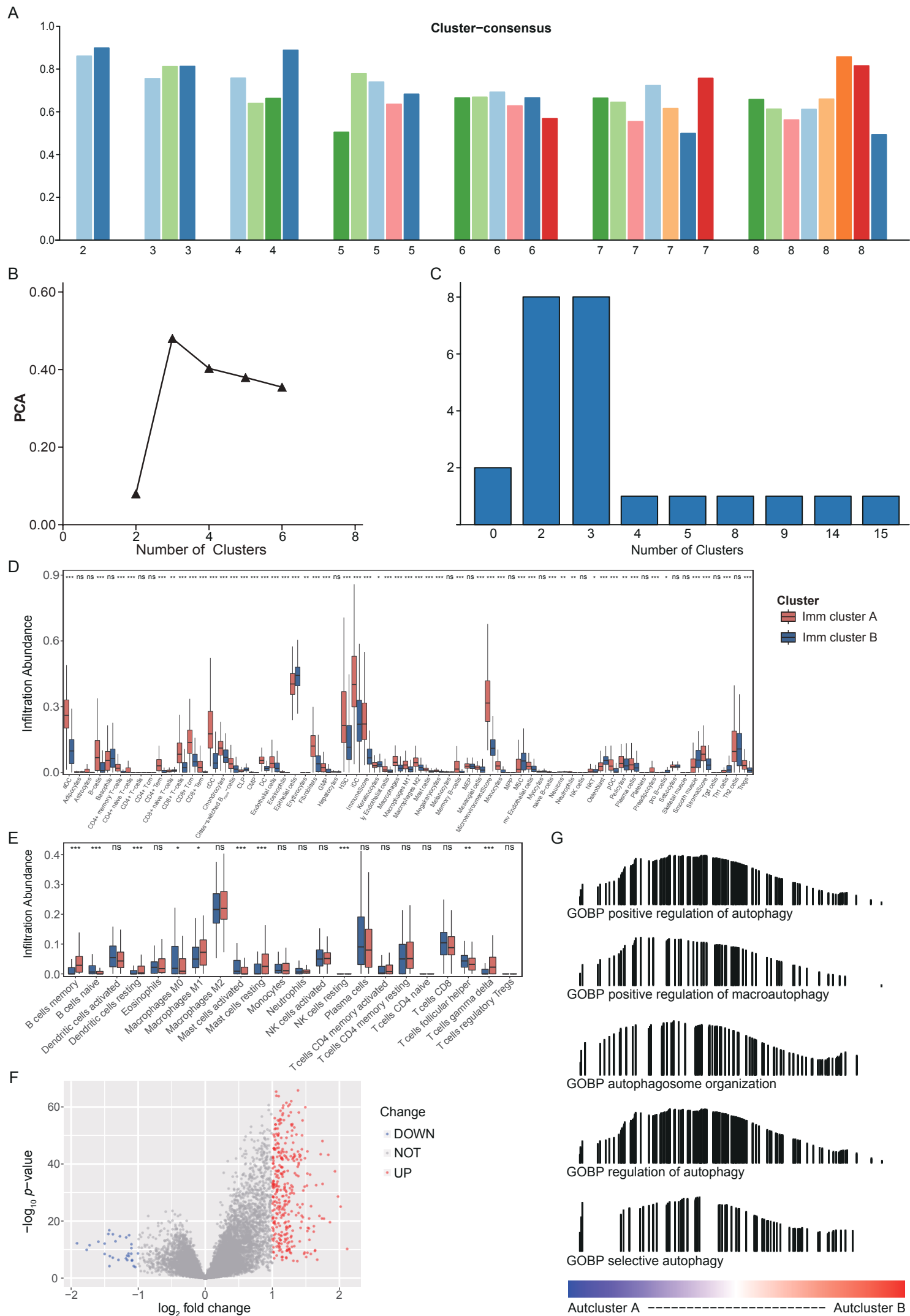

Supplement: Supplementary file 2 [file DataSheet_2.pdf]

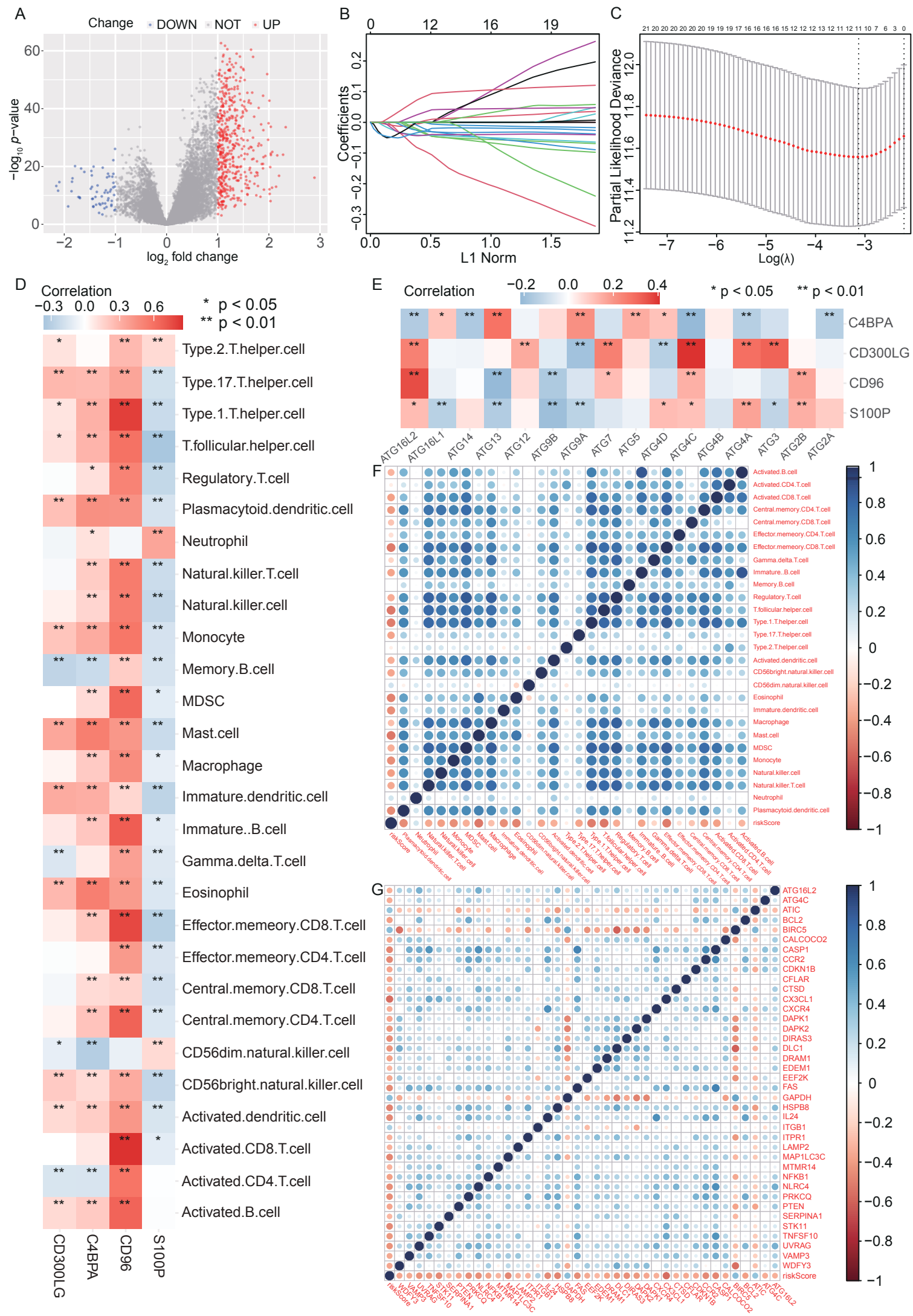

Supplement: Supplementary file 3 [file DataSheet_3.pdf]

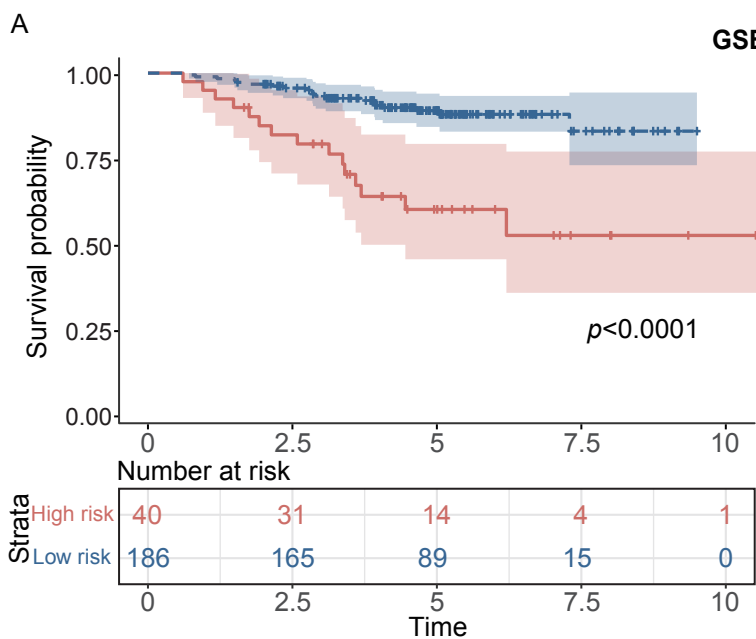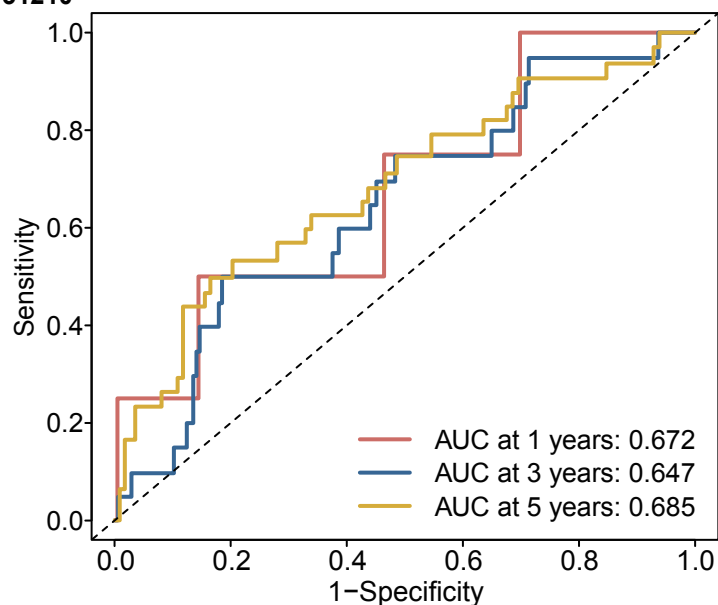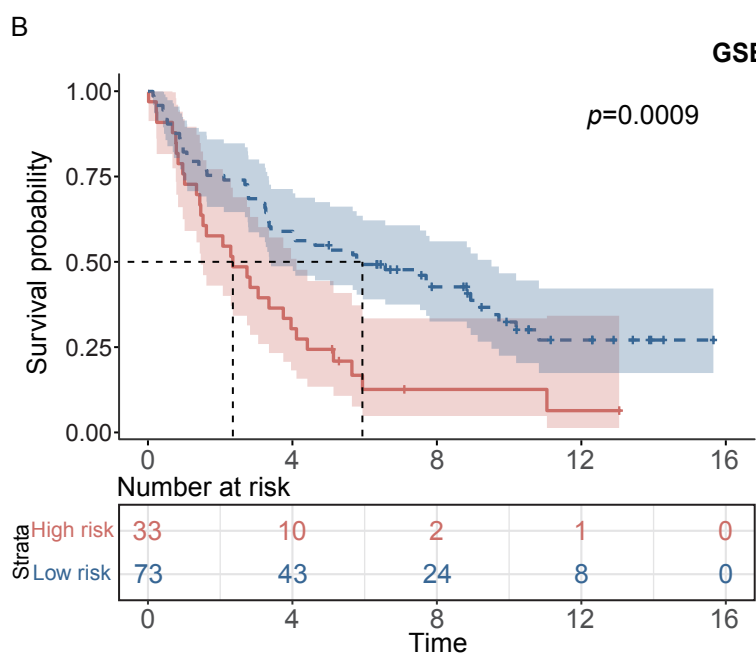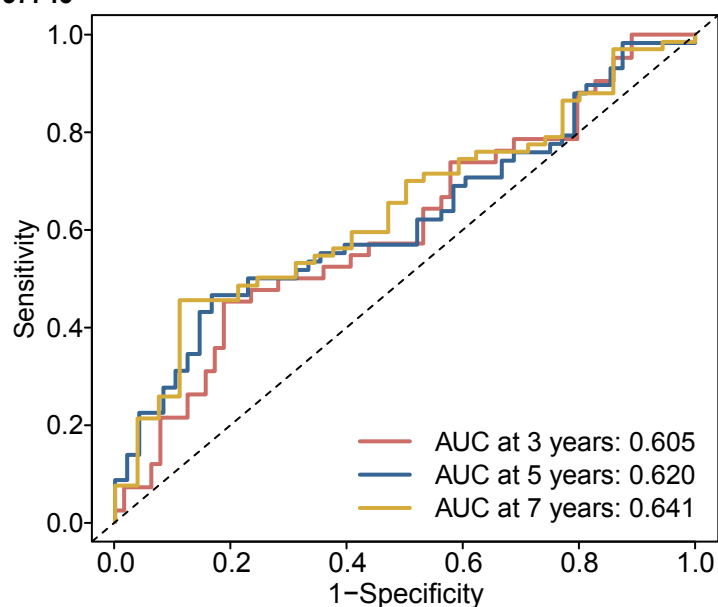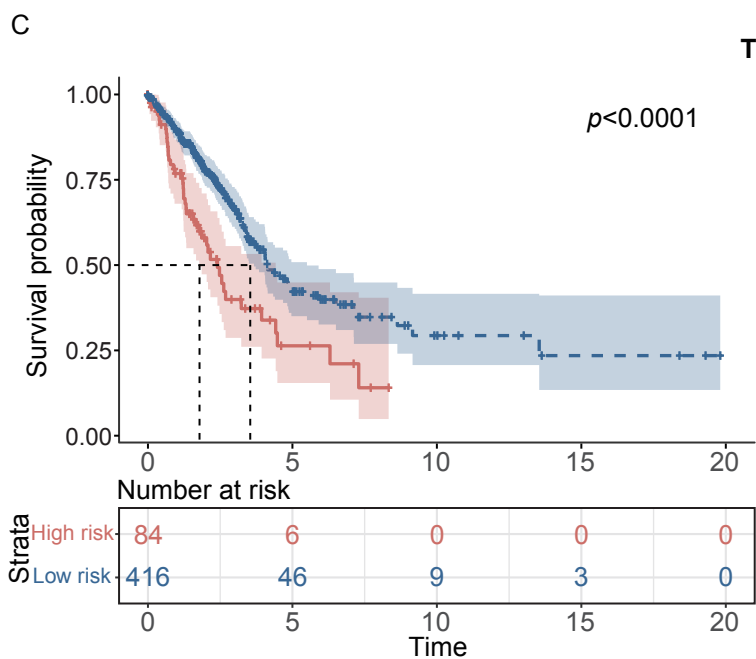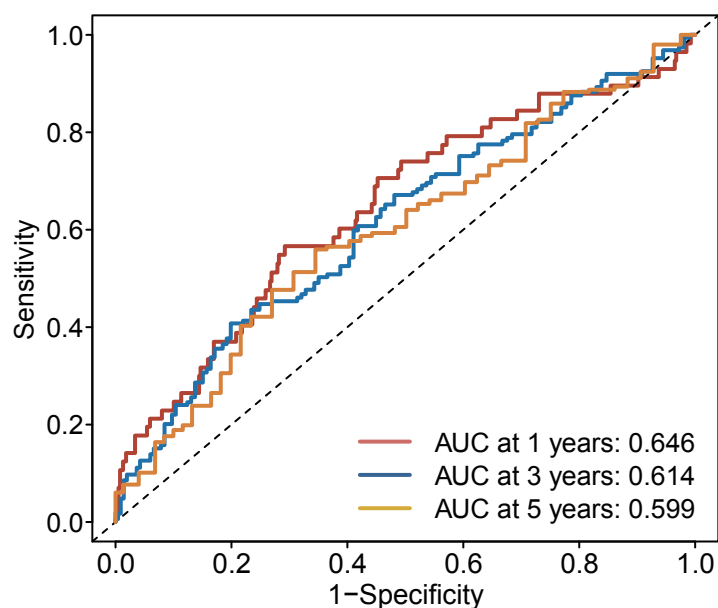

Supplement: Supplementary file 4 [file DataSheet_4.pdf]

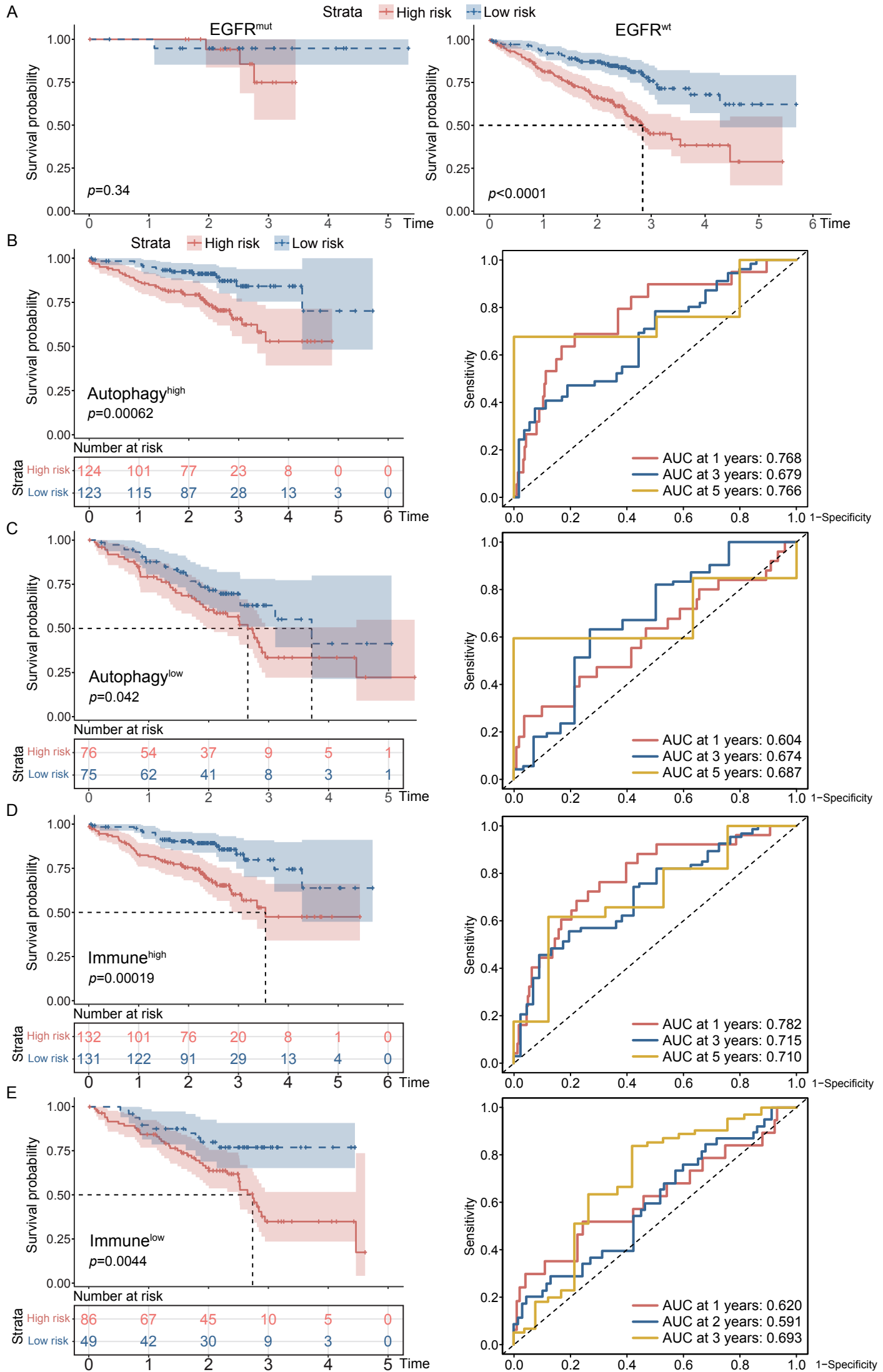

Supplement: Supplementary file 5 [file DataSheet_5.pdf]

A

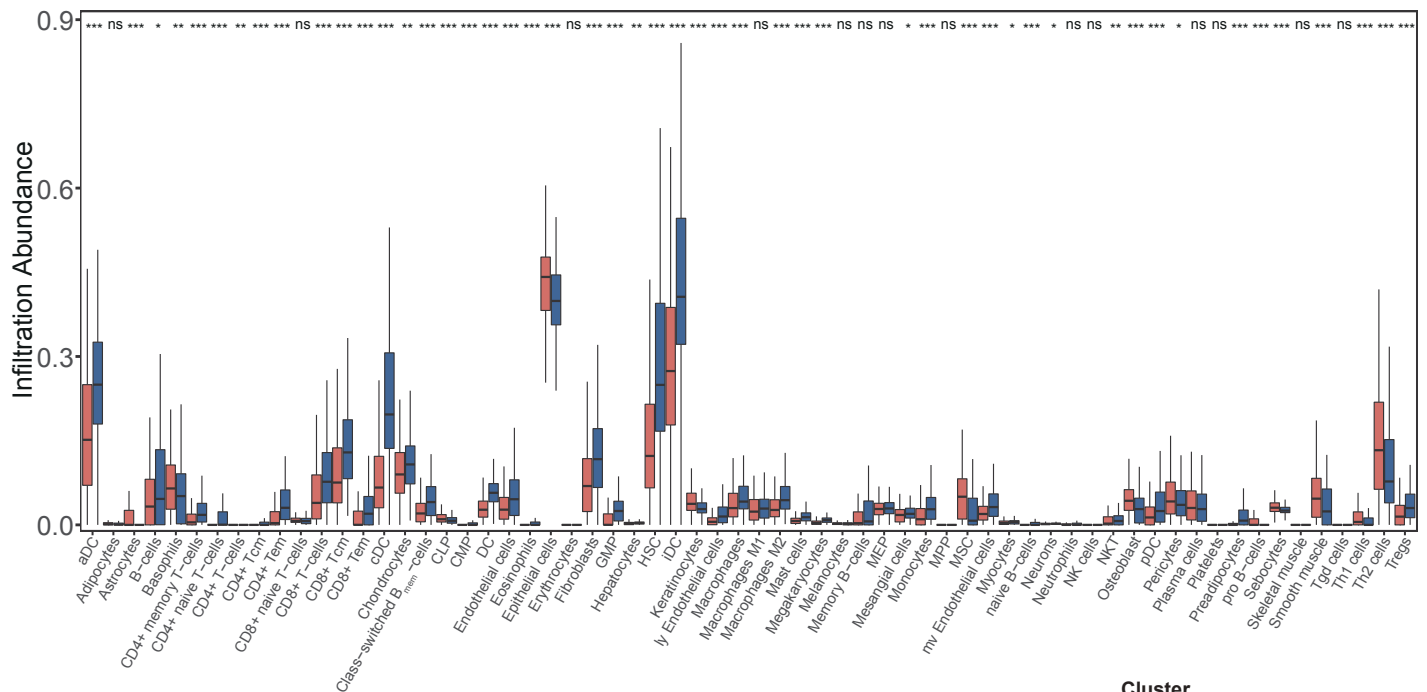

B

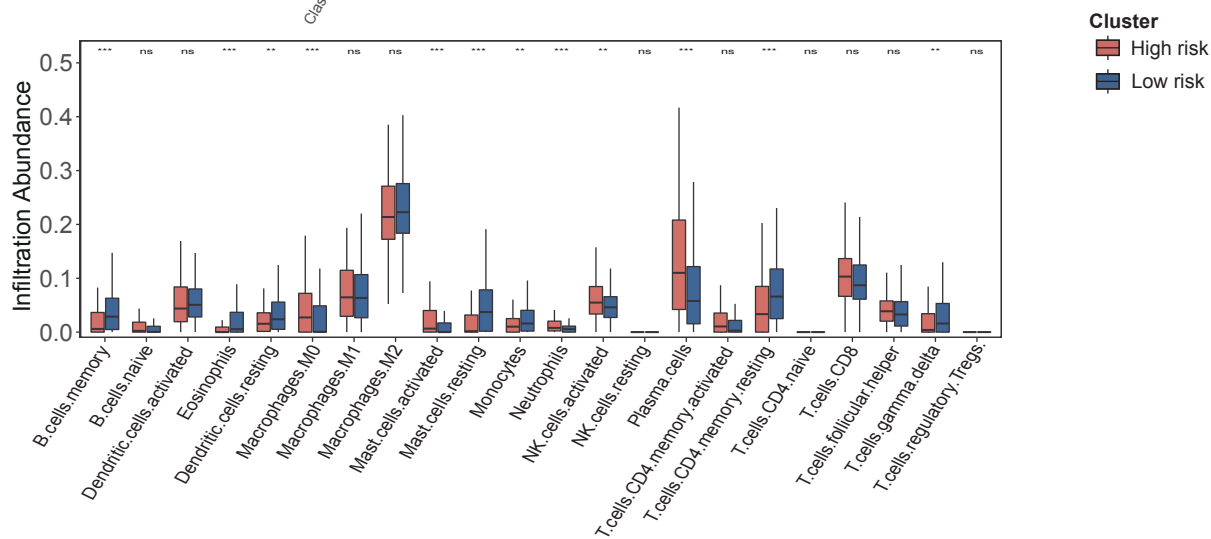

C

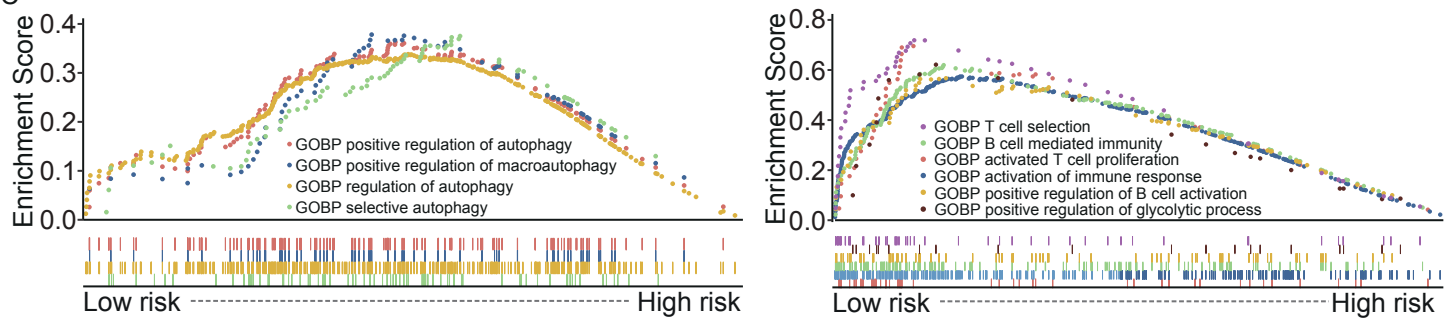

D

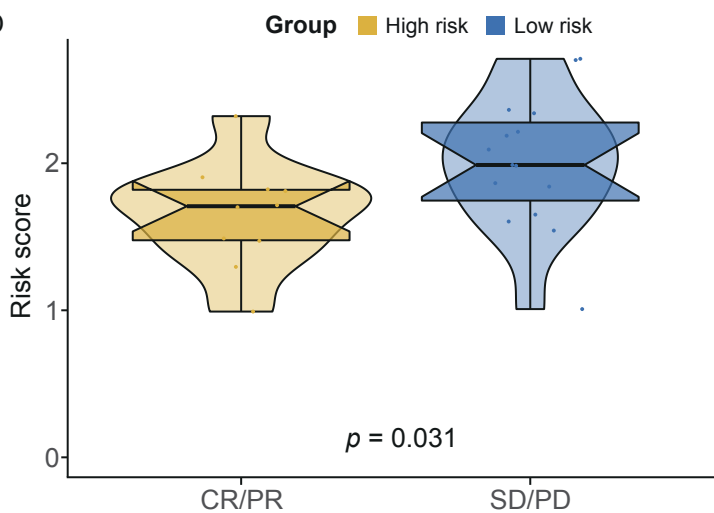

E

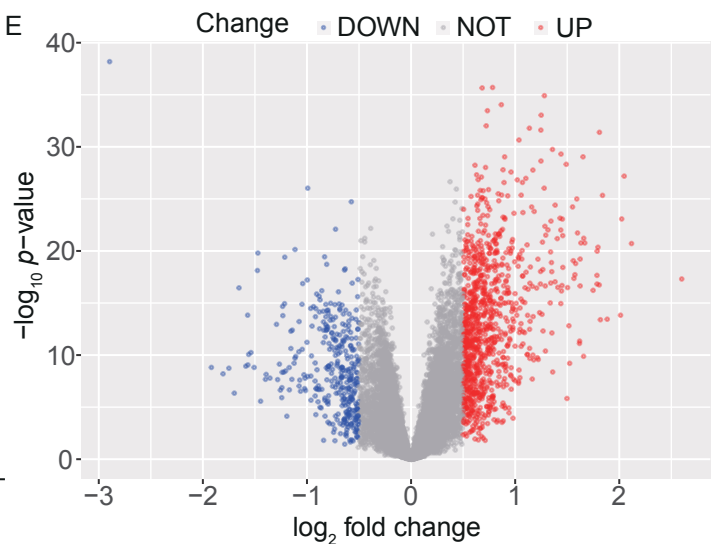

Supplement: Supplementary file 6 [file DataSheet_6.pdf]

A

CD300LG

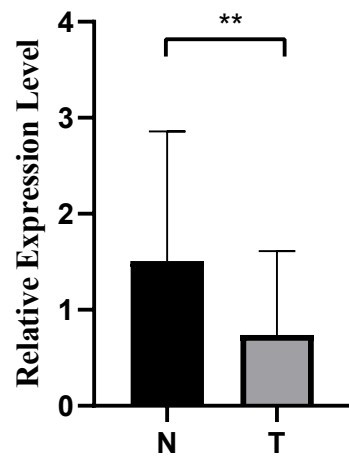

B

CD96

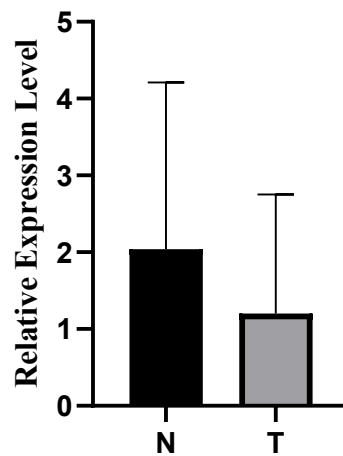

C

C4BPA

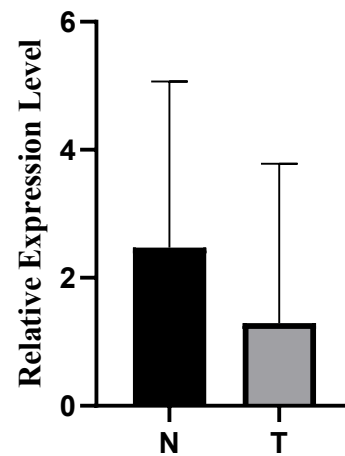

D

S100P

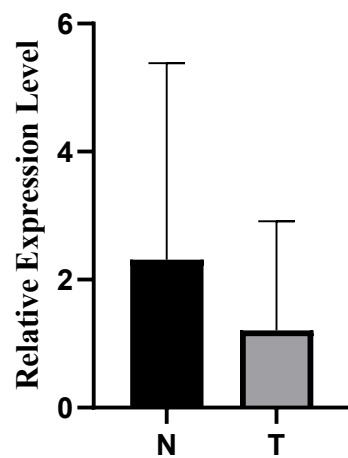

E

S100P

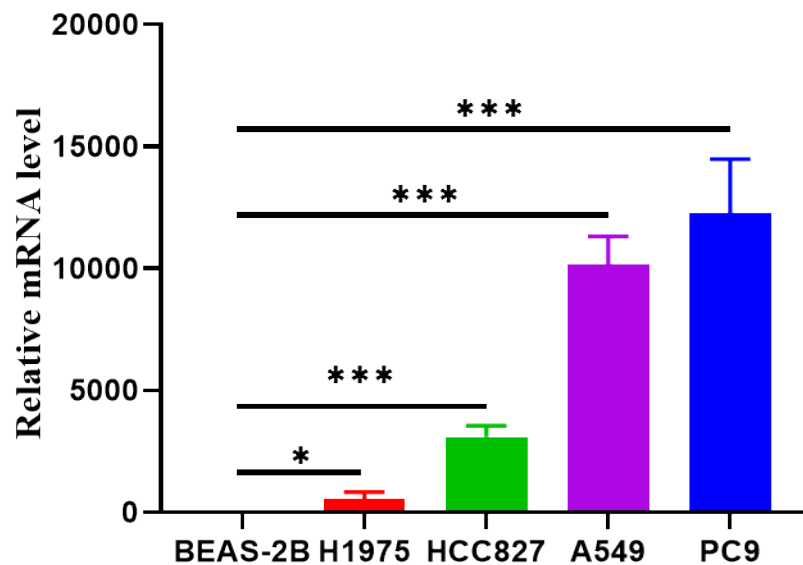

Supplement: Supplementary file 7 [file DataSheet_7.pdf]
